# Frizzled class receptor 5 contributes to ovarian cancer chemoresistance through aldehyde dehydrogenase 1A1

**DOI:** 10.1186/s12964-024-01585-y

**Published:** 2024-03-27

**Authors:** Yuhong Xia, Shan Wang, Yu Sun, Wei Wang, Shijie Chang, Zhongbo Zhang, Chenghai Zhao

**Affiliations:** 1 Department of Pathophysiology, College of Basic Medical Science, China Medical University, Shenyang, Liaoning Province 110122 P.R. China; 2 Department of Biomedical Engineer, School of Intelligent Medicine, China Medical University, Shenyang, Liaoning Province 110122 P.R. China; 3 Department of Pancreatic and Biliary Surgery, The First Hospital of China Medical University, Shenyang, Liaoning Province 110001 P.R. China

**Keywords:** Chemoresistance, Stemness, Homologous recombination repair, DNA-damaging therapy, Ovarian cancer

## Abstract

**Background:**

Chemoresistance is associated with tumor relapse and unfavorable prognosis. Multiple mechanisms underlying chemoresistance have been elucidated, including stemness and DNA damage repair. Here, the involvement of the WNT receptor, FZD5, in ovarian cancer (OC) chemoresistance was investigated.

**Methods:**

OC cells were analyzed using in vitro techniques including cell transfection, western blot, immunofluorescence and phalloidin staining, CCK8 assay, colony formation, flowcytometry, real-time PCR, and tumorisphere formation. Pearson correlation analysis of the expression levels of relevant genes was conducted using data from the CCLE database. Further, the behavior of OC cells in vivo was assessed by generation of a mouse xenograft model.

**Results:**

Functional studies in OC cells showed that FZD5 contributes to epithelial phenotype maintenance, growth, stemness, HR repair, and chemoresistance. Mechanistically, FZD5 modulates the expression of ALDH1A1, a functional marker for cancer stem-like cells, in a β-catenin-dependent manner. ALDH1A1 activates Akt signaling, further upregulating RAD51 and BRCA1, to promote HR repair.

**Conclusions:**

Taken together, these findings demonstrate that the FZD5-ALDH1A1-Akt pathway is responsible for OC cell survival, and targeting this pathway can sensitize OC cells to DNA damage-based therapy.

**Supplementary Information:**

The online version contains supplementary material available at 10.1186/s12964-024-01585-y.

## Background

Ovarian cancer (OC) is a highly lethal disease. Incidence and mortality of OC both rank eighth in women [1]. Most patients have poor prognosis due to being diagnosed at a later stage. The mortality-to-incidence ratio in OC is greater than 0.6 [2]. Treatment of OC includes surgery, chemotherapy, targeted therapy, and several evolving therapies such as folate receptor targeting and immunotherapy. Platinum-based chemotherapy is widely used for the treatment of OC, but many patients have resistance to these drugs [3]. Enhancing sensitivity to chemotherapy can prevent or delay OC relapse, and improve patient survival.

Homologous recombination (HR) is an important mechanism for repair of DNA double-strand breaks (DSBs) [4, 5]. Various factors, including the recombinase, RAD51, and breast cancer susceptibility gene 1/2 (BRCA1/2), play crucial roles in this process. As DSBs seriously threaten genome integrity and stability, HR defects, for example BRCA1/2 mutations, increase the risk of tumorigenesis; however, HR-deficient tumors are sensitive to DNA-damaging agents. In contrast, HR-proficient tumors have enhanced HR repair capacity, and are more tolerant to DNA damage. Therefore, inhibiting HR repair is a feasible strategy to sensitize HR-proficient tumors to DNA-damaging drugs, such as cisplatin and PARP inhibitors (PARPi) [6, 7].

Cancer stem-like cells (CSCs) are a small subpopulation of cancer cells with self-renewal characteristics, as well as some other features of normal stem cells. CSCs are generally resistant to DNA damage, which facilitates their survival on exposure to chemotherapy and radiotherapy [8, 9]. In OC, ALDH-positive (ALDH^+^) cells exhibit stem-like characteristics and chemoresistance [10, 11]. Targeting ALDH1A1, a member of the ALDH1 family, suppresses OC initiation and growth, as well as sensitizing OC cells to platinum-based chemotherapeutic drugs [12–14]. ALDH1A1 downregulation or inhibition induces DNA damage, indicating that ALDH1A1 contributes to both stemness and DNA damage repair [14, 15].

Cisplatin is the first FDA-approved platinum compound for cancer treatment. Currently, cisplatin is often used in combination with other drugs to treat a variety of human tumors, including OC, lung cancer, breast cancer, and head and neck squamous cell carcinoma [16]. Cisplatin is able to crosslink with the purine bases on the DNA, causing DNA damage and cell apoptosis. Various biological processes are involved in cisplatin resistance, such as drug efflux, DNA damage repair, epithelial-mesenchymal transition, and autophagy [17–21]. In the present study, we identified ALDH1A1 as a downstream effector of the FZD5-β-catenin pathway. Further, we demonstrate that FZD5 promotes stemness and HR repair via ALDH1A1 and Akt, thereby inducing OC cell chemoresistance.

## Methods

### Cell culture and transfection

OVCAR3, SKOV3 and CAOV3 cells were cultured in DMEM (Hyclone) containing 10% fetal bovine serum (FBS) in a humidified incubator with 5% CO2 at 37℃. These cells were transfected with shFZD5 lentiviruses (GV112/hU6- MCS-CMV-Puromycin, Genechem, China) to stably knockdown FZD5 expression. Puromycin (2 µg/ml, Sigma) was used to select cells at 48 h after infection. The target sequence for shFZD5-1 is 5′-CGGCATCTTCACGCTGCTCTA-3′, for shFZD5-2 is 5′-GGCCACCTTCCTCATCGACAT-3′, and for control is 5′-TTCTCCGAACGTGTCACGT-3′. FZD5-knockdowned cells was transfected with ALDH1A1 overexpression plasmid (GV219/CMV-MCS-SV40-Neomycin, Genechem, China) according to the manufacturer’s instructions.

### Western blot

Cells are lysed using RIPA lysis buffer containing 1% PMSF for 1 h on ice. The cell lysates were centrifuged at 12,000×g for 40 min at 4℃. The protein concentration of the supernatant was determined using a BCA assay. Total protein lysate (30 µg) was separated by gel electrophoresis on a 12% SDS-PAGE gel and transferred to a PVDF membrane. The membranes were blocked in Tris-buffered saline-Tween 20 (TBST) containing 5% skimmed milk for 2 h at room temperature. The membranes were incubated with primary antibodies at 4℃ overnight. Subsequently, the membranes were washed 3 times with TBST and then incubated with secondary antibodies for 2 h at room temperature. The chemiluminescence (ECL) detection system (Tanon 5200, Shanghai, China) was applied for the imaging of the target protein expression. The following primary antibodies were used: FZD5 (Cell Signaling Technology, #5266, USA, 1:1000), E-cadherin (Cell Signaling Technology, #3195, USA, 1:1000), ALDH1A1 (Santa Cruz, sc-374,076, USA, 1:500), BRCA1 (Cell Signaling Technology, #9010, USA, 1:1000), RAD51 (Abcam, ab133534, UK, 1:1000), active β-catenin (Cell Signaling Technology, #8814, USA, 1:1000), Akt (Cell Signaling Technology, #9272, USA, 1:1000), pAkt (Cell Signaling Technology, #9271, USA, 1:1000), pErk1/2 (Cell Signaling Technology, #4370, USA, 1:1000), and pJnk1/2 (Cell Signaling Technology, #9251, USA, 1:1000).

### Immunohistochemistry

25 OC tissues and 7 normal ovarian tissues were collected from Tumor hospital of China medical university with the informed consent of the patients. The use of the specimens for research was approved by Institutional Research Ethics Committee of Tumor hospital of China Medical University (KY20230905). The tissues were fixed in 4% paraformaldehyde, embedded in paraffin and then sliced into 4 μm sections. Xylene and gradient alcohol were used to deparaffinize and hydrate, respectively. 3% H2O2 was used to eliminate endogenous peroxidase activity. Sections were incubated with anti-FZD5 antibody (Abcam, ab75234, UK, 1:200) overnight at 4 °C and corresponding second antibody at 37 °C for 30 min. Subsequently, sections were stained with DAB. Finally, sections were examined under a microscope (Leica, USA, DM2500 LED).

### Survival analysis

Correlation of FZD5 with overall survival (OS) and progression-free survival (PFS) in OC was analyzed using GSE26193 database at Kaplan-Meier Plotter website (https://kmplot.com/analysis/). Correlation of ALDH1A1 with OS and PFS in OC was analyzed using GSE26712 database at Kaplan-Meier Plotter website.

### Immunofluorescence

Cells were inoculated on slides in 24-well plates at 40–50% confluence and grown in an incubator at 37℃ for 24 h, fixed with paraformaldehyde for 20 min and washed with PBS three times. Next, cells were permeabilized with 0.5% Triton X-100 for 5 min at room temperature, blocked with 5% donkey serum for 1 h at room temperature and incubated with primary antibody at 4℃ overnight; the following primary antibodies were used: E-cadherin (Cell Signaling Technology, #3195, USA, 1:200), EPCAM (Immunoway, YM6053, USA, 1:100), γH2AX antibody (Cell Signaling Technology, #9718, USA, 1:400), and active β-catenin (Cell Signaling Technology, #8814, USA, 1:800). Subsequently, slides were incubated with fluorescent-labeled secondary antibody for 2 h. Nuclei were stained with DAPI in the dark and visualized using a laser scanning confocal microscope.

### Phalloidin staining

Cells were fixed with 4% paraformaldehyde at room temperature for 10 min. Slides were washed three times with PBS, and incubated with 0.5% Triton X-100, then treated with TRITC-conjugated Phalloidin solution (YEASEN, Shanghai, China) at room temperature for 30 min. Nuclei were stained with DAPI. Cell morphology was visualized by laser scanning confocal microscopy.

### Correlation analysis of gene expression

Gene expression at mRNA levels was investigated in a panel of human OC cell lines using data from the CCLE database. Correlation analysis of gene expression levels, including those of *FZD5*, *CDH1*, *EPCAM*, *ALDH1A1*, *ALDH1A2*, *ALDH1A3*, *ALDH1B1*, *ALDH1L1*, and *ALDH1L2*, was performed using the Pearson statistic.

### Cell proliferation assay

Cells (OVCAR3: 10^3^; SKOV3: 2 × 10^3^; CAOV3: 10^3^) were resuspended in 100 µl medium, seeded into 96-well plates, and incubated for the indicated length of time. Then, cells were treated with 10 µl Cell Counting Kit-8 reagent (CCK8, Dojindo Molecular Technologies, Japan) and incubated at 37℃ for 4 h. Absorbance values at 450 nm were measured at different time points using a microplate reader (Bio-Rad Laboratories, USA).

### Colony formation assay

Cells (2 × 10^3^) were seeded and cultured in 3.5 cm culture dishes for 2 weeks until visible colonies formed. Cells were then fixed with 4% paraformaldehyde for 20 min. Colonies were washed with PBS, stained with 1% crystal violet for 20 min, counted, and photographed.

### Cell cycle assay

Cells (1 × 10^6^) were collected, washed with PBS, and fixed with 70% ethanol at 4℃ overnight. Then, cells were treated with 500 µl PI/RNaSeA staining solution (KeyGEN BioTECH, China) for 1 h at room temperature in the dark. Samples were analyzed using a FACS Calibur Flow Cytometer (BD, USA).

### Real-time PCR

RNAiso Plus (Takara, China) was added to collected cells to extract total RNA, following the standard instructions. After RNA quantification, reverse transcription was performed with 1 µg RNA using a cDNA synthesis Kit (Takara, RR047A, China). Then, real-time PCR was performed using a TB Green™ Premix Ex Taq II kit (Takara, China) and an ABI PRISM 7300 Sequence Detection system (Applied Biosystems, USA). *GAPDH* was used as an internal control. Gene expression was analyzed by the 2^−ΔΔ^ Ct method. The primers used are listed in Table 1.

**Table 1:** Primers for real-time PCR

Genes	Primers (5′–3′)
FZD5-forward FZD5-reverse ALDH1A1-forward ALDH1A1-reverse RAD51-forward RAD51-reverse BRCA1-forward BRCA1-reverse GAPDH-forward GAPDH-reverse	TCCTCTGCATGGATTACAACC GACACTTGCACACGAACG GACAATGCTGTTGAATTTGCAC AAGGATATACTTCTTAGCCCGC TGGCAGTGGCTGAGAGGTATGG GGTCTGGTGGTCTGTGTTGAACG AGGTCCAAAGCGAGCAAGAGAATC CTGTGGGCATGTTGGTGAAGGG CAGGAGGCATTGCTGATGAT GAAGGCTGGGGCTCATTT

### ALDH^+^ cell subpopulation assay

Cells (1 × 10^6^) were suspended in 1 ml ALDEFLUOR™ Assay Buffer (STEMCELL Technologies, USA) and 5 µl ALDEFLUOR™ Reagent was added in each tube. Then, the mixture was divided into two equal parts: diethylaminobenzaldehyde was added into one part as negative control. After incubation at 37℃ for 45 min, cells were re-suspended in 500 µl ALDEFLUOR™ Assay Buffer. Samples were analyzed using a FACS Calibur Flow Cytometer (BD, USA).

### Extreme limiting dilution analysis

Cells (100, 50, 25, 10/well) were cultured in complete MammoCult™ Human Medium (STEMCELL Technologies, USA) in 96-well ultra-low-attachment plates (Corning, USA) for 7 days. Stemness was evaluated using a tool available at https://bioinf.wehi.edu.au/software/elda/.

### HR repair assay

Cells were transiently co-transfected with DR-GFP and I-SceI plasmids (Genechem, China) using Lipofectamine 3000 (Invitrogen), according to the manufacturer’s instructions, then cultured for 3 days. The percentage of GFP^+^ cells was analyzed using a FACS Calibur Flow Cytometer (BD, USA).

### Cytotoxicity assay

After inoculation into 96-well plates at a density of 5000/well overnight, cells were treated with cisplatin or olaparib at different concentrations for 48 h. Then, cells were treated with 10 µl CCK8 reagent (Dojindo, Japan) and incubated at 37℃ for 4 h. Absorbance values at 450 nm were measured using a microplate reader (Bio-Rad Laboratories, USA).

### Tumorisphere toxicity assay

Cells (5 × 10^3^) were treated with or without cisplatin (5 µM) in complete MammoCult™ Human Medium (STEMCELL Technologies, USA) in 6-well ultra-low-attachment plates (Corning, USA) for 7 days. Tumor spheres were counted in five randomly selected fields.

### Apoptosis assay

Cells (1 × 10^6^) were collected and washed with PBS and an Annexin V-PE/7- AAD Apoptosis Kit (KeyGEN BioTECH, China) used to analyze cell apoptosis. Cells were incubated in 500 µL binding buffer with 5 µL 7-AAD at room temperature in the dark for 15 min. The number of apoptotic cells was measured using a FACS Calibur Flow Cytometer (BD, USA).

### Mice and in vivo analysis

Female BALB/c nude mice (5–6 weeks old, 18–20 g) were purchased from Weitong Lihua (Beijing, China) and maintained in the specific pathogen free-grade facility of the Department of Animal Experimentation of China Medical University. All studies involving mice were approved by the Animal Ethics Committee of China Medical University (CMU2021249). After resuspension in 100 µl PBS, 1 × 10^6^ OC cells were injected subcutaneously into the lateral flanks of the mice (*n* = 5 per group). Tumor volume was calculated using the formula: *V* = ½ × length × width^2^. Tumor length and width were measured every 3 days using a Vernier caliper. Four weeks after inoculation, mice were euthanized and the tumors harvested. For in vivo chemo-sensitivity assay, mice were administered cisplatin (5 mg/kg, intraperitoneal injection) for five consecutive days, once the tumor volume reached approximately 50 mm^3^.

### Statistical analysis

Data are presented as mean ± SD and were analyzed using GraphPad Prism 9. Differences were analyzed by two-sided Student’s t-test, or one-way or two-way ANOVA. *P* < 0.05 was considered significant.

## Results

### FZD5 maintains an epithelial phenotype in OC cells

**Fig. 1:**
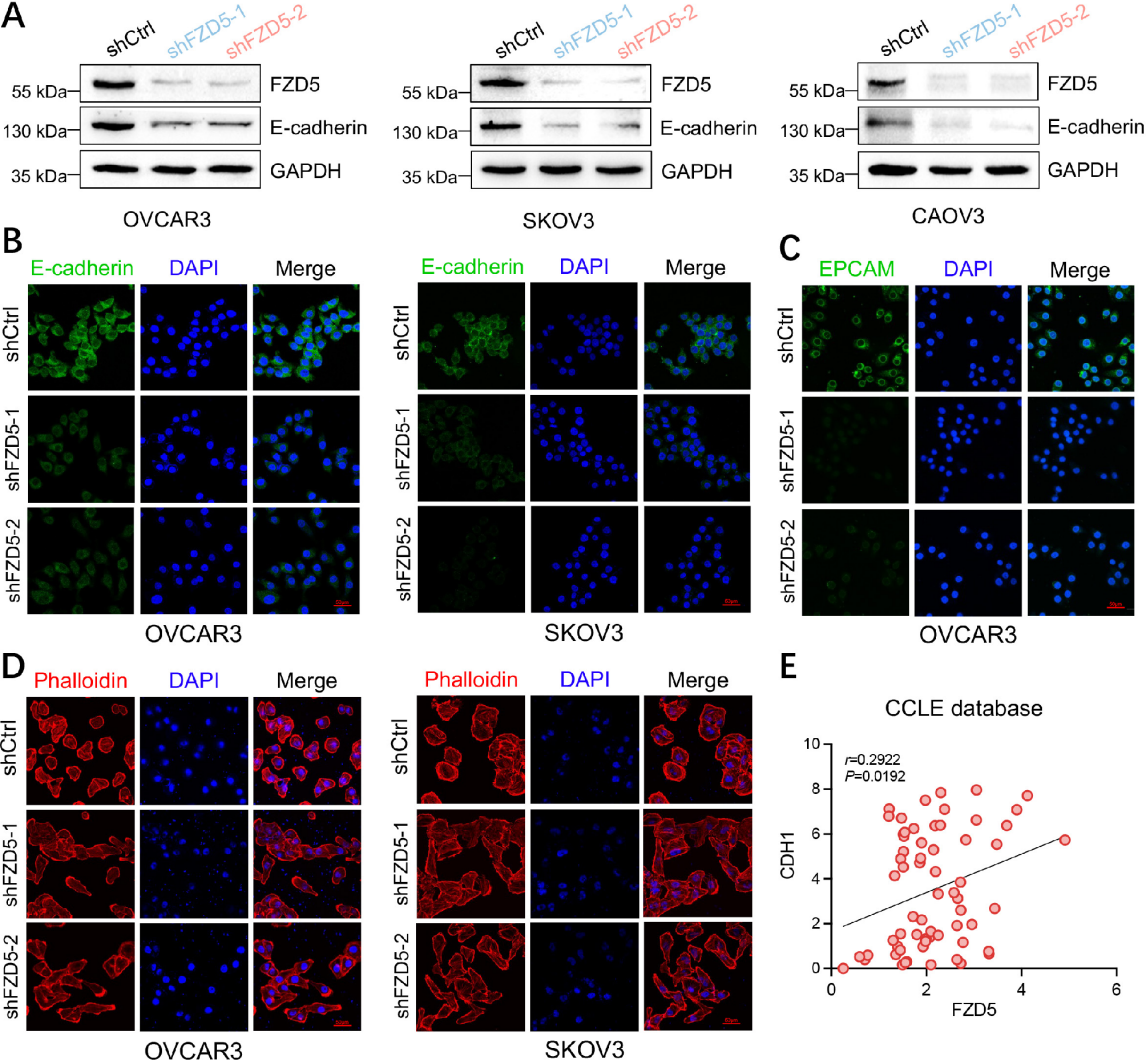
FZD5 maintains an epithelial phenotype in OC cells. **A** FZD5 and E-cadherin expression in OC cells detected by western blot. **B** E-cadherin expression in OC cells detected by immunofluorescence staining; scale bar: 50 μm. **C** EPCAM expression in OC cells detected by immunofluorescence staining; scale bar: 50 μm. **D** OC cells stained with phalloidin; scale bar: 50 μm. **E** CCLE database analysis showing positive correlation of *FZD5* and *CDH1* mRNA levels in OC.

FZD5 expression in human specimens was detected by Immunohistochemistry staining. 22 of 25 OC tissues exhibited positive staining compared to 2 of 7 normal ovarian tissues (Additional file 1, Fig. S1). Higher FZD5 expression was associated with shorter overall survival and progression-free survival in OC (Additional file 1, Fig. S2). Our group previously found that FZD5 is implicated in the maintenance of an epithelial phenotype in gastric and bladder cancers [22, 23]. To explore whether FZD5 functions similarly in OC, three epithelial OC cell lines were stably transfected with FZD5 shRNA lentiviruses. FZD5 knockdown reduced the expression of E-cadherin, a key marker of epithelial phenotype (Fig. 1A, B). Further, FZD5 knockdown also downregulated the expression of EPCAM, a marker of both epithelial and stem-like phenotypes (Fig. 1C). Consistently, FZD5 knockdown induced a morphologic change from epithelial-like to mesenchymal-like (Fig. 1D). Moreover, CCLE database analysis indicated that *FZD5* mRNA levels were positively correlated with those of *CDH1* (the gene encoding E-cadherin) and *EPCAM* in OC (Fig. 1E; Additional file 1, Fig. S3).

### FZD5 promotes OC cell growth

**Fig. 2:**
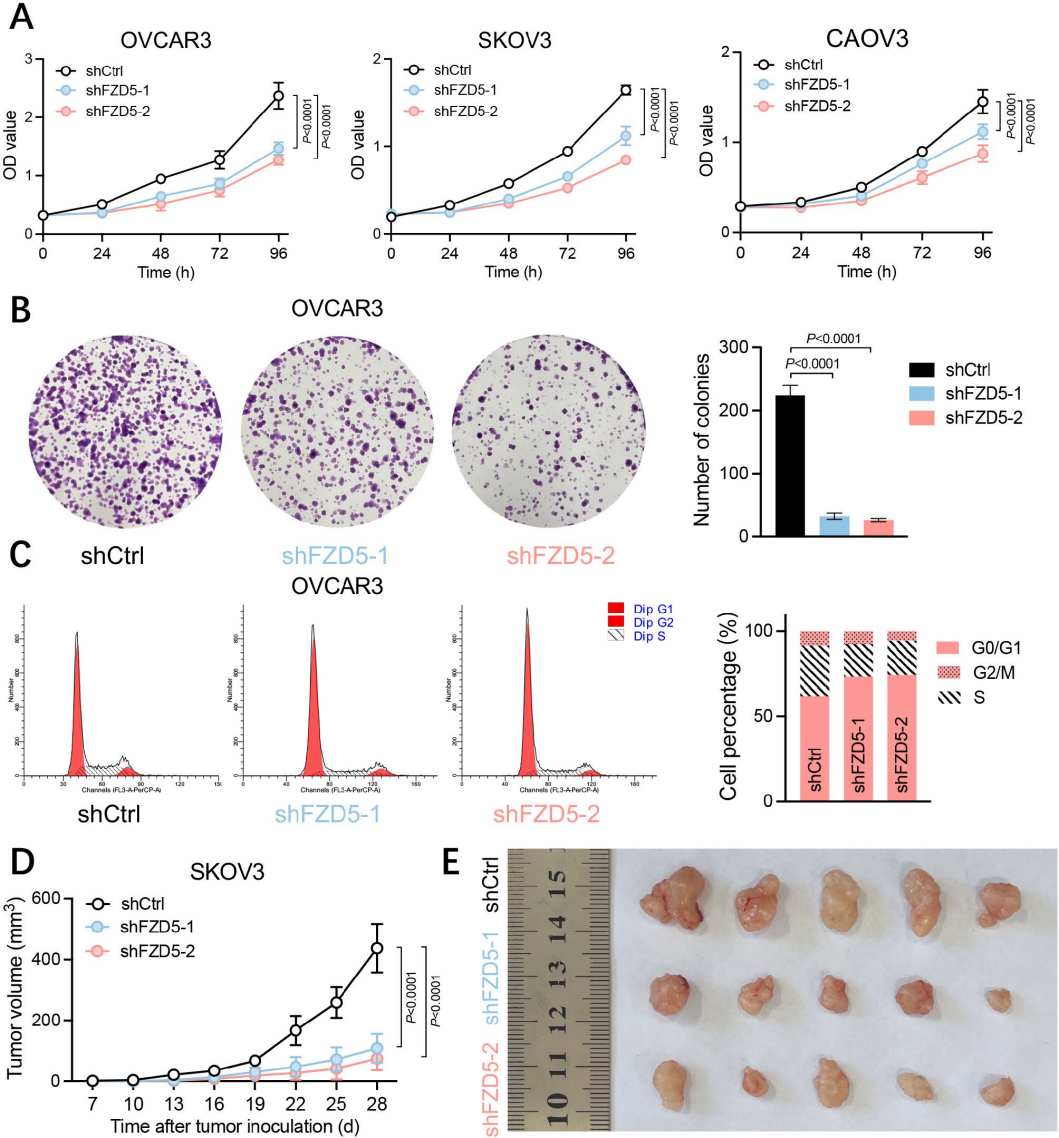
FZD5 promotes OC cell growth. **A** Cell viability analyzed by CCK8 assay (mean ± SD, *n* = 3). **B** Growth of OVCAR3 cells determined by colony formation assay (mean ± SD, *n* = 3). **C** Cell cycle of OVCAR3 cells analyzed by flow cytometry. **D, E** Growth curves of xenograft tumors from SKOV3 cells (mean ± SD, *n* = 5)

The epithelial phenotype is associated with proliferation of OC cells [24]. Therefore, we next investigated the role of FZD5 in OC cell growth. As shown by CCK8 and colony formation tests, FZD5 knockdown slowed cell proliferation, and reduced the formation of cell colonies (Fig. 2A, B; Additional file 1, Fig. S4). Further, FZD5 knockdown decreased the fraction of cells in S-phase, while increasing that in G1-phase, indicating that FZD5 affects the entry of cells into S-phase (Fig. 2C). Next, SKOV3 cells were inoculated into immune-deficient mice, to further evaluate the effect of FZD5 on cell growth. Our data demonstrate that xenograft tumors from cells with FZD5 knocked down grew more slowly than those from control cells (Fig. 2D, E). Together, these results demonstrate that FZD5 facilitates OC growth.

### FZD5 induces stem-like properties in OC cells

**Fig. 3:**
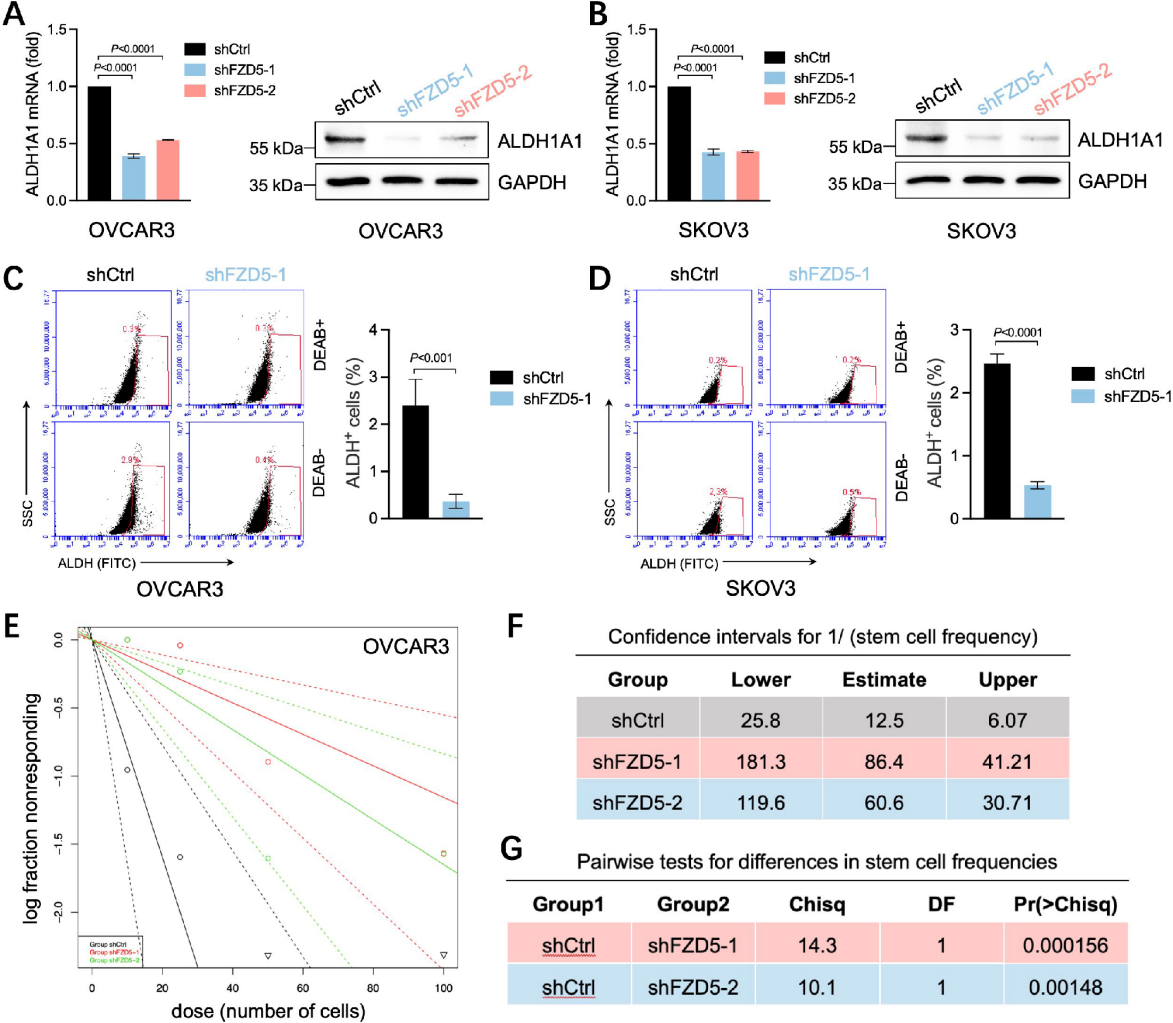
FZD5 induces stem-like properties in OC. **A, B** ALDH1A1 expression in OC cells detected by real-time PCR and western blot (mean ± SD, *n* = 3). **C, D** Proportion of ALDH^+^ cells detected by flow cytometry (mean ± SD, *n* = 3). **E–G** Stemness of OVCAR3 cells analyzed by extreme limiting dilution mammosphere formation assay

Our previous study revealed that FZD5 modulates ALDH^+^ stem-like cells in breast cancer [25]. To identify the ALDH1 family member targeted by FZD5 in OC, we conducted correlation analysis using data from the CCLE database. Among five members of the ALDH1 family, including *ALDH1A1*, *ALDH1A2*, *ALDH1A3*, *ALDH1B1*, *ALDH1L1*, and *ALDH1L2*, levels of *FZD5* were positively correlated with those of *ALDH1A1* (Additional file 1, Fig. S5). Next, we examined ALDH1A1 expression in OC cells. As expected, FZD5 knockdown led to downregulation of ALDH1A1 at both the mRNA and protein levels (Fig. 3A, B). Consistent with this finding, FZD5 knockdown diminished the ALDH^+^ stem-like subpopulation (Fig. 3C, D). Further, extreme limiting dilution analysis demonstrated that FZD5 knockdown attenuated OC cell stemness (Fig. 3E-G).

### FZD5 enhances HR repair capacity

**Fig. 4:**
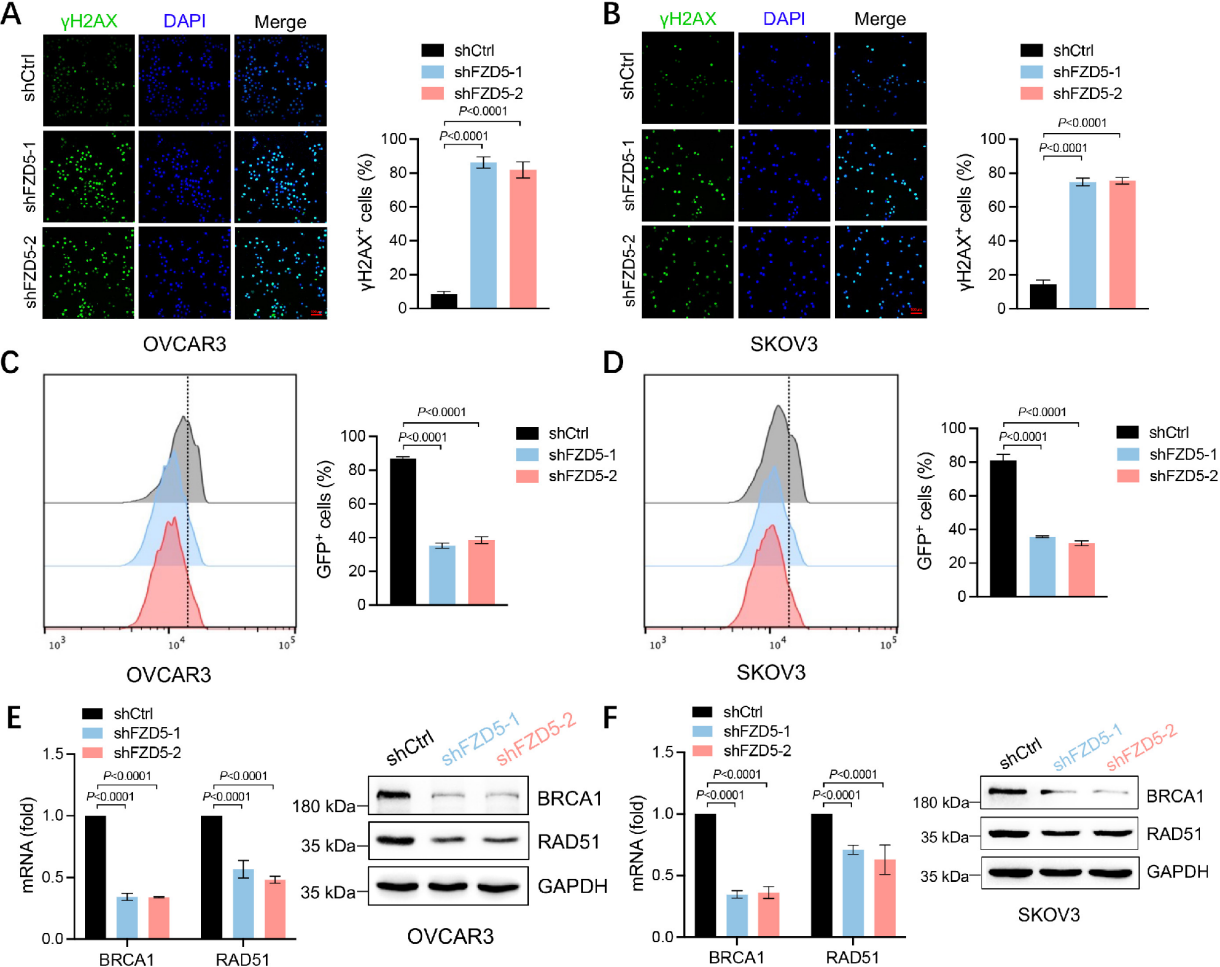
FZD5 enhances HR repair capacity. **A, B** γ-H2AX expression in OC cells detected by immunofluorescence staining 24 h after treatment with cisplatin (5 µM) (mean ± SD, *n* = 3); scale bar: 100 μm. **C, D** HR repair of OC cells analyzed by flow cytometry (mean ± SD, *n* = 3). **E, F** BRCA1 and RAD51 expression in OC cells detected by real-time PCR and western blot (mean ± SD, *n* = 3)

ALDH1A1 is involved in DNA damage repair in OC [14, 15, 26]; therefore, we next assessed whether FZD5 plays a role in this process. After treatment with the DNA-damaging drug, cisplatin, cells with FZD5 knocked down displayed more γ-H2AX staining than control cells, demonstrating that FZD5 knockdown impaired DNA repair following DNA damage (Fig. 4A, B). To assess whether FZD5 modulates HR repair, OC cells were co-transfected with I-SceI and DR-GFP plasmids. The fraction of GFP^+^ cells in cells with FZD5 knocked down was significantly lower than that in control cells, indicating that FZD5 knockdown disrupted HR repair (Fig. 4C, D). Mechanistically, FZD5 knockdown reduced the expression of RAD51 and BRCA1, two key factors involved in HR repair (Fig. 4E, F).

### FZD5 endows OC cells with chemoresistance

**Fig. 5:**
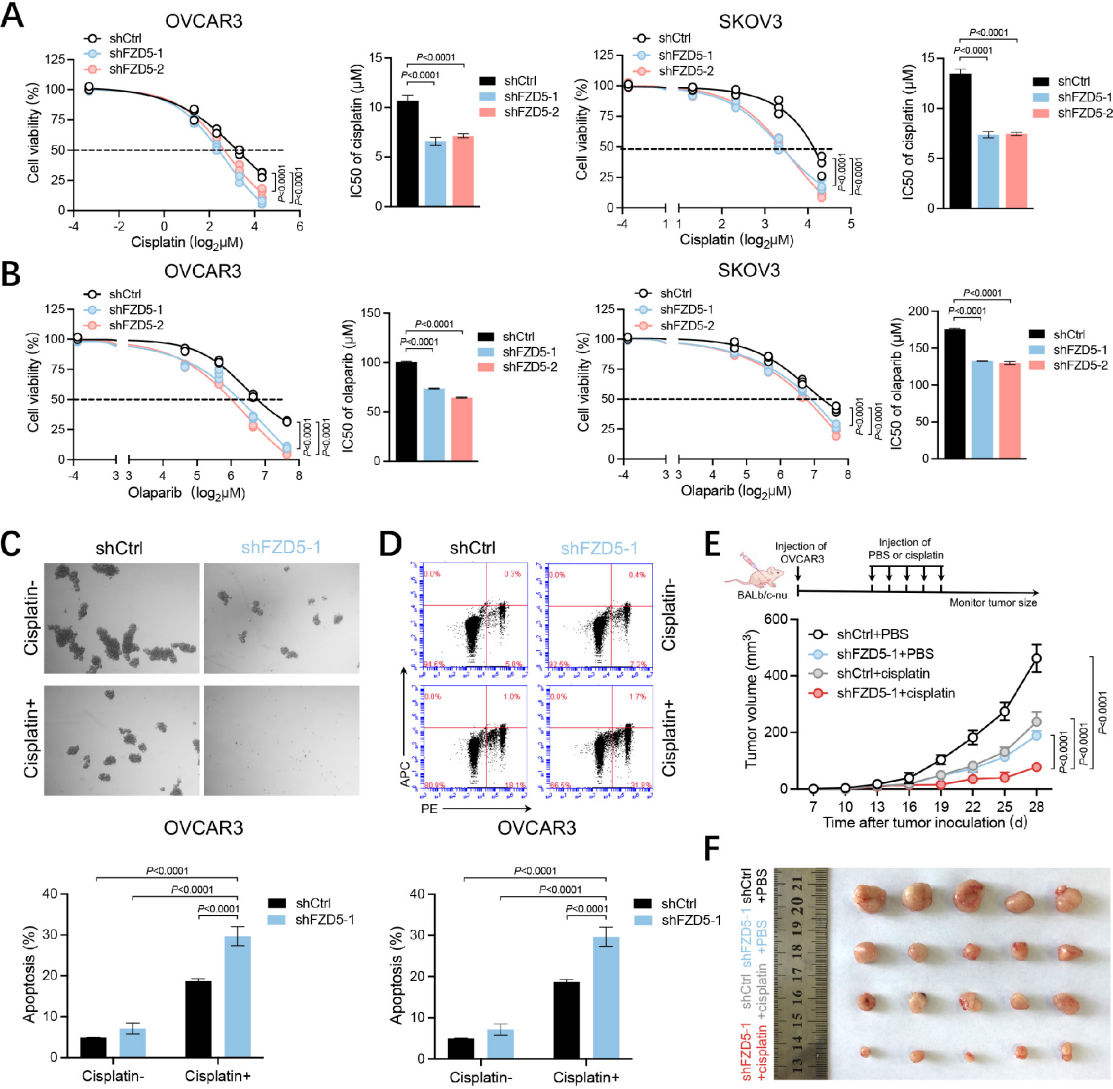
FZD5 endows OC cells with chemoresistance. **A** Viability of OC cells 48 h after treatment with cisplatin analyzed by CCK8 assay (mean ± SD, *n* = 3). **B** Viability of OC cells 48 h after treatment with olaparib analyzed by CCK8 assay (mean ± SD, *n* = 3). **C** Mammosphere formation of OVCAR3 cells treated with or without cisplatin (10 µM) (mean ± SD, *n* = 3). **D** Apoptosis of OVCAR3 cells treated with or without cisplatin (10 µM) detected by flow cytometry (mean ± SD, *n* = 3). **E, F** Growth curves of OVCAR3-xenograft tumors treated with or without cisplatin (5 mg/kg) (mean ± SD, *n* = 5)

Both stemness and HR repair are related to therapeutic resistance. Therefore, the role of FZD5 in chemoresistance was subsequently evaluated. Relative to control cells, cells with FZD5 knocked down exhibited higher sensitivity to cisplatin (Fig. 5A, Additional file 1, Fig. S6A). HR-deficient cells are sensitive to PARPi due to synthetic lethality. Indeed, FZD5 knockdown sensitized OC cells to olaparib (Fig. 5B, Additional file 1, Fig. S6B). Further, FZD5 knockdown worked with cisplatin treatment to inhibit mammosphere formation, indicating that targeting FZD5 also can increase the sensitivity of ovarian CSCs to DNA-damaging agents (Fig. 5C). Moreover, FZD5 knockdown remarkably increased cisplatin-induced apoptosis (Fig. 5D). Finally, FZD5 knockdown also worked with cisplatin treatment to suppress the growth of xenograft tumors (Fig. 5E, F).

### ALDH1A1 is a downstream effector of FZD5 in OC

**Fig. 6:**
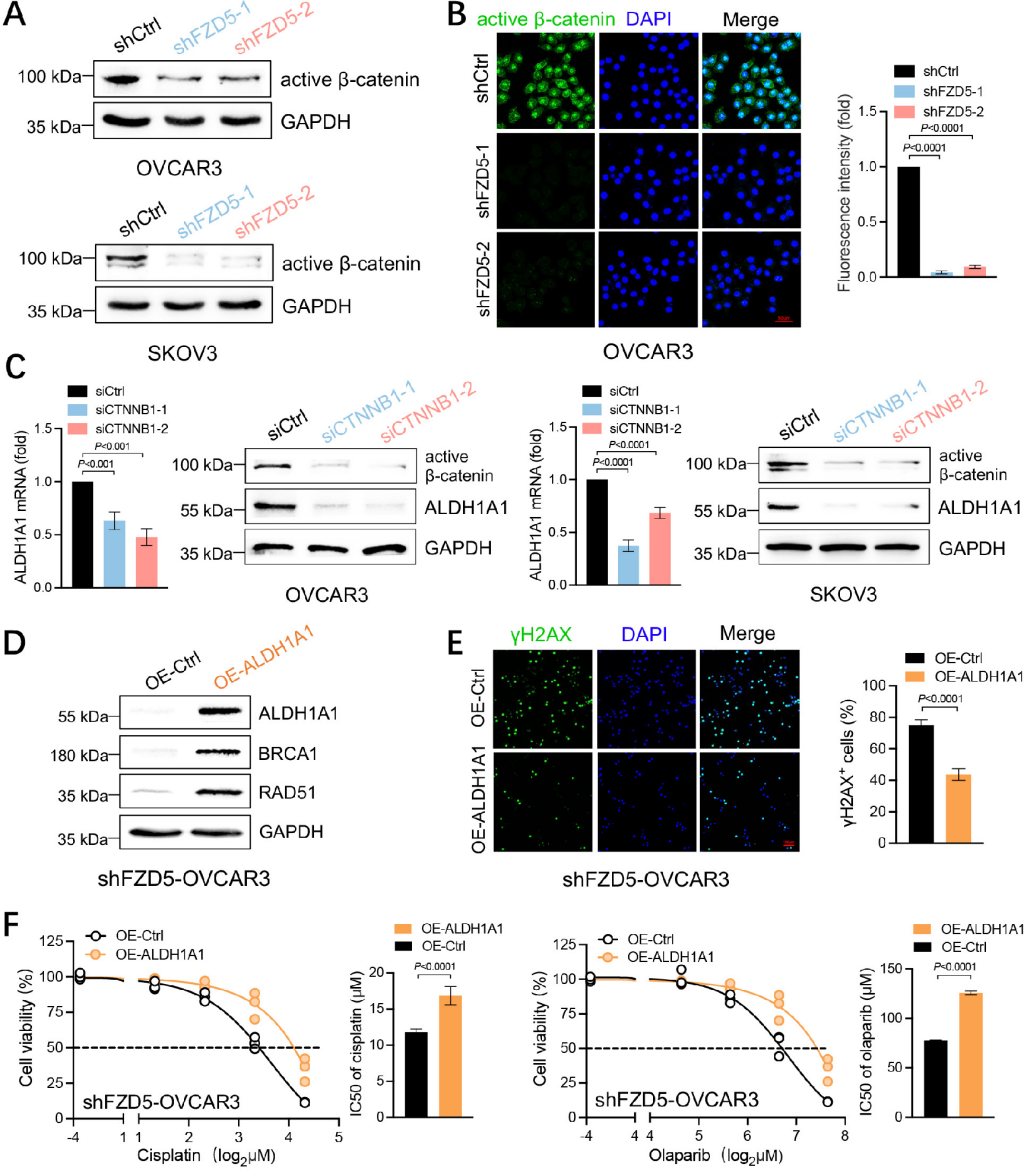
ALDH1A1 is a downstream effector of FZD5. **A** Active β-catenin expression in OC cells detected by western blot. **B** Active β-catenin expression in OVCAR3 cells detected by immunofluorescence staining; scale bar: 50 μm. **C** ALDH1A1 and active β-catenin expression in OC cells detected by real-time PCR and western blot (mean ± SD, *n* = 3). **D** ALDH1A1, BRCA1, and RAD51 expression in OVCAR3 cells with FZD5 knocked down detected by western blot. **E** γ-H2AX expression in OVCAR3 cells with FZD5 knocked down detected by immunofluorescence staining 24 h after treatment with cisplatin (5 µM) (mean ± SD, *n* = 3); scale bar: 100 μm. **F** Viability of OVCAR3 cells with FZD5 knocked down 48 h after treatment with cisplatin or olaparib analyzed by CCK8 assay (mean ± SD, *n* = 3)

FZD5 mediates both β-catenin-dependent and -independent pathways [27]. In OC cells, FZD5 knockdown downregulated the expression of active β-catenin, indicating that FZD5 can activate the β-catenin pathway (Fig. 6A, B; Additional file 1, Fig. S7). Treatment with XAV939, a β-catenin inhibitor, sensitized OC cells to cisplatin (Additional file 1, Fig. S8). To determine whether FZD5 modulation of ALDH1A1 is dependent on β-catenin, we transfected OC cells with siRNAs targeting *CTNNB1*, the gene encoding β-catenin. CTNNB1 knockdown downregulated ALDH1A1 at both the mRNA and protein levels (Fig. 6C). To determine whether ALDH1 mediates FZD5-induced HR repair and chemoresistance, ALDH1A1 was overexpressed in cells with FZD5 knocked down. Our data demonstrate that ALDH1 overexpression restored the expression of RAD51 and BRCA1, as well as DNA damage repair capacity (Fig. 6D, E). Furthermore, cells with FZD5 knocked down and ALDH1A1 overexpression regained resistance to cisplatin and olaparib (Fig. 6F). Finally, higher *ALDH1A1* expression was associated with shorter overall survival and progression-free survival in OC (Additional file 1, Fig. S9).

### The FZD5-ALDH1A1 pathway activates akt signaling

**Fig. 7:**
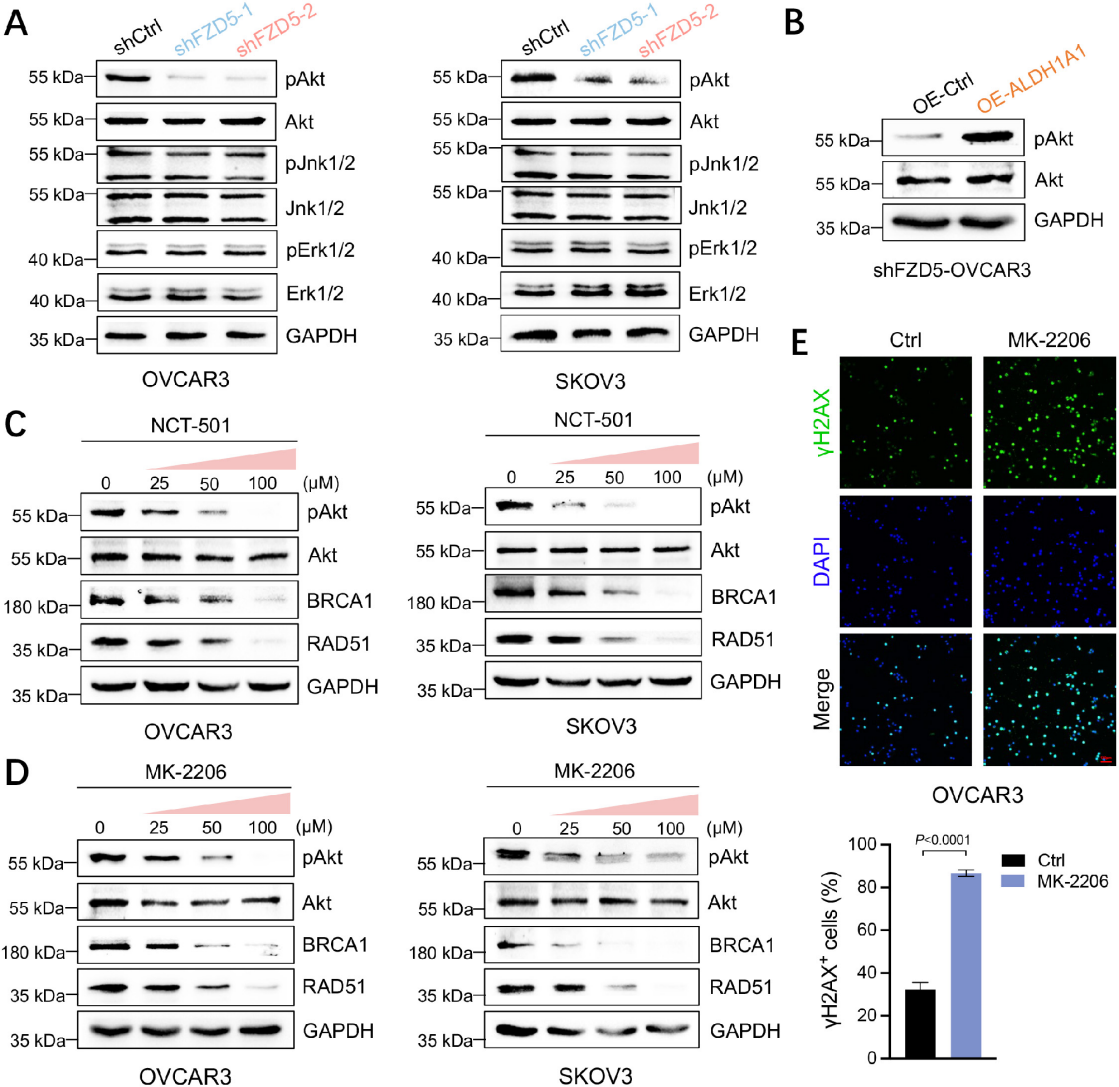
The FZD5-ALDH1A1 pathway activates Akt signaling. **A** pAkt, Akt, pJnk, Jnk, pErk, and Erk expression in OC cells detected by western blot. **B** pAkt and Akt expression in OVCAR3 cells with FZD5 knocked down detected by western blot. **C** pAkt, Akt, BRCA1, and RAD51 expression in OC cells 48 h after treatment with NCT-501 detected by western blot. **D** pAkt, Akt, BRCA1, and RAD51 expression in OC cells 48 h after treatment with MK-2206 detected by western blot. **E** γ-H2AX expression in OVCAR3 cells detected by immunofluorescence staining 24 h after treatment with cisplatin (5 µM) (mean ± SD, *n* = 3); scale bar: 100 μm. **F** Schematic showing the FZD5-ALDH1A1-Akt pathway

To elucidate the signaling downstream of the FZD5-ALDH1A1 pathway in OC cells, we analyzed the phosphorylation of Akt, Jnk, and Erk. FZD5 knockdown repressed the phosphorylation of Akt, but had no effect on that of Jnk or Erk (Fig. 7A). Overexpression of ALDH1A1 in cells with FZD5 knocked down restored the expression of phosphorylated Akt (pAkt) (Fig. 7B). Furthermore, treatment of OC cells with NCT-501, an ALDH1A1 inhibitor, reduced pAkt, BRCA1 and RAD51 expression in a dose-dependent manner (Fig. 7C). Next, to investigate whether Akt signaling is related to HR repair, OC cells were treated with the Akt inhibitor, MK-2206. Treatment with MK-2206 decreased the expression of BRCA1 and RAD51, as well as DNA damage repair capacity in OC cells (Fig. 7D, E). These results indicate that the FZD5-ALDH1A1-Akt pathway is responsible for HR repair in OC cells (Fig. 7F).

## Discussion

Frizzled (FZD) molecules belong to the seven-transmembrane G protein-coupled receptor (GPCR) superfamily, which contain a highly conserved cysteine-rich domain (CRD). Extracellular WNT molecules bind to the CRD to trigger both the canonical β-catenin pathway and various β-catenin-independent pathways. There are 10 members in the human FZD family, FZD1–FZD10, and FZD proteins are involved in many human diseases, affecting almost every aspect of tumor development, including tumorigenesis, growth, metastasis, chemoresistance, and recurrence [27]. In this study, we provide the first report of the role of FZD5 in OC chemoresistance.

We found that FZD5 sustains the epithelial phenotype of OC cells through modulation of E-cadherin and EPCAM. It is established that E-cadherin functions as a negative regulator of epithelial-mesenchymal transition (EMT), thus blocking tumor cell invasion and migration; however, E-cadherin has also been demonstrated to facilitate tumor progression [28, 29]. In OC, E-cadherin promotes cell proliferation and chemoresistance [24, 30]. EPCAM is an epithelial and stem-like dual marker, and EPCAM-high/positive OC cells are more tolerant to chemotherapy than those with an EPCAM-low/negative phenotype [31].

There are two types of CSC in breast cancer, mesenchymal-like and epithelial-like [32, 33]. Mesenchymal-like CSCs are CD24^−^CD44^+^, quiescent, and motile, due to high expression of mesenchymal markers, such as vimentin. Epithelial CSCs are ALDH^+^ and EPCAM^+^, proliferative, and immotile, because of high expression of E-cadherin. Ovarian CSCs exhibit similar heterogeneity [34]. A subpopulation of ovarian CSCs express CD44 and TGFβ1, and are characterized by EMT [35, 36]. As a negative regulator of TGFβ1 signaling, SMAD7 maintains the epithelial phenotype of ovarian CSCs, and promotes their colonization of metastatic sites by inducing mesenchymal-epithelial transition [37].

Our study reveals that FZD5 upregulates E-cadherin and EPCAM, as well as ALDH1A1, in OC, indicating that FZD5 maintains OC CSCs in an epithelial-like state. Consistently, FZD5 promotes cell growth, stemness, and chemoresistance. Further, we found that ALDH1A1 induces HR repair via Akt, which upregulates the HR factors, RAD51 and BRCA1. ALDH1A1 was also shown to activate Akt signaling in non-small cell lung cancer and esophageal squamous cell carcinoma [38, 39]; however, the mechanism by which ALDH1A1 induces Akt phosphorylation is unknown.

## Conclusions

**Fig. 8:**
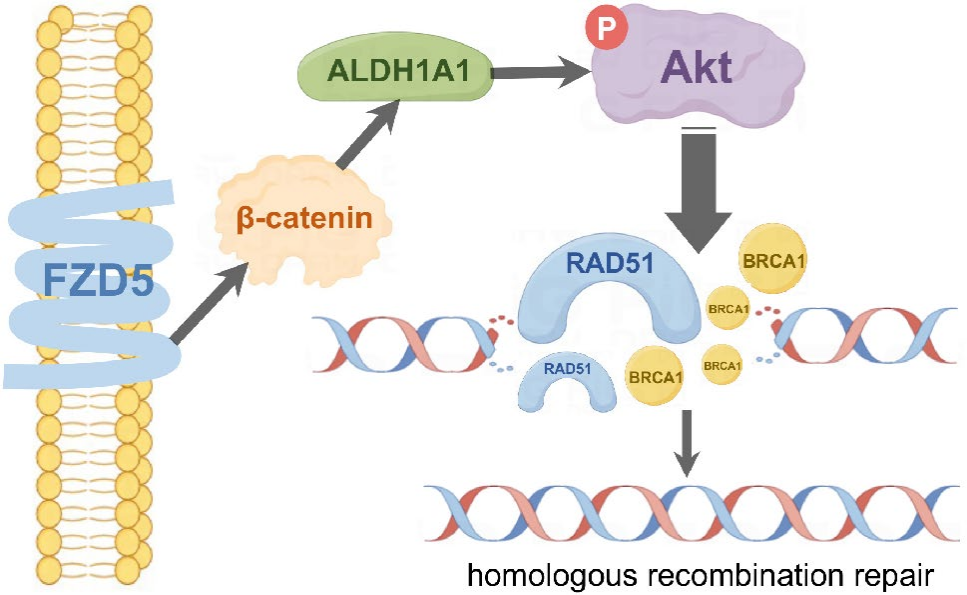
FZD5-ALDH1A1-Akt pathway in OC. FZD5 upregulates ALDH1A1 expression in a β-catenin-dependent manner. ALDH1A1 activates Akt signaling to enhance HR repair by upregulating RAD51 and BRCA1.

In summary, FZD5 induces stemness and HR repair, thereby endowing OC cells with resistance to DNA-damaging agents. Mechanistically, FZD5 promotes ALDH1A1 expression in a β-catenin-dependent manner. ALDH1A1 then activates Akt signaling, upregulating RAD51 and BRCA1 to enhance HR repair. Taken together, these findings demonstrate that the FZD5-ALDH1A1-Akt pathway contributes to OC chemoresistance, and that targeting this pathway has potential to sensitize OC cells to DNA damage-based therapy (Fig. 8).

### Electronic supplementary material

Below is the link to the electronic supplementary material.

Supplementary Material 1

## Data Availability

The datasets used and/or analysed during the current study are available from the corresponding author on reasonable request.

## Abbreviations

CRD: Cysteine rich domain
CSC: Cancer stem like cells
DSB: Double strand breaks
EMT: Epithelial mesenchymal transition
GPCR: G protein coupled receptor
HR: Homologous recombination
OC: Ovarian cancer
