# Supplementary material for: Frizzled class receptor 5 contributes to ovarian cancer chemoresistance through aldehyde dehydrogenase 1A1
Source: Cell Commun Signal. 2024 Mar 27;22:194. doi: 10.1186/s12964-024-01585-y (PMC10967208; doi:10.1186/s12964-024-01585-y)
Supplement: Supplementary file 1 — Supplementary Material 1 [file 12964_2024_1585_MOESM1_ESM.docx]

**Supplementary figures**


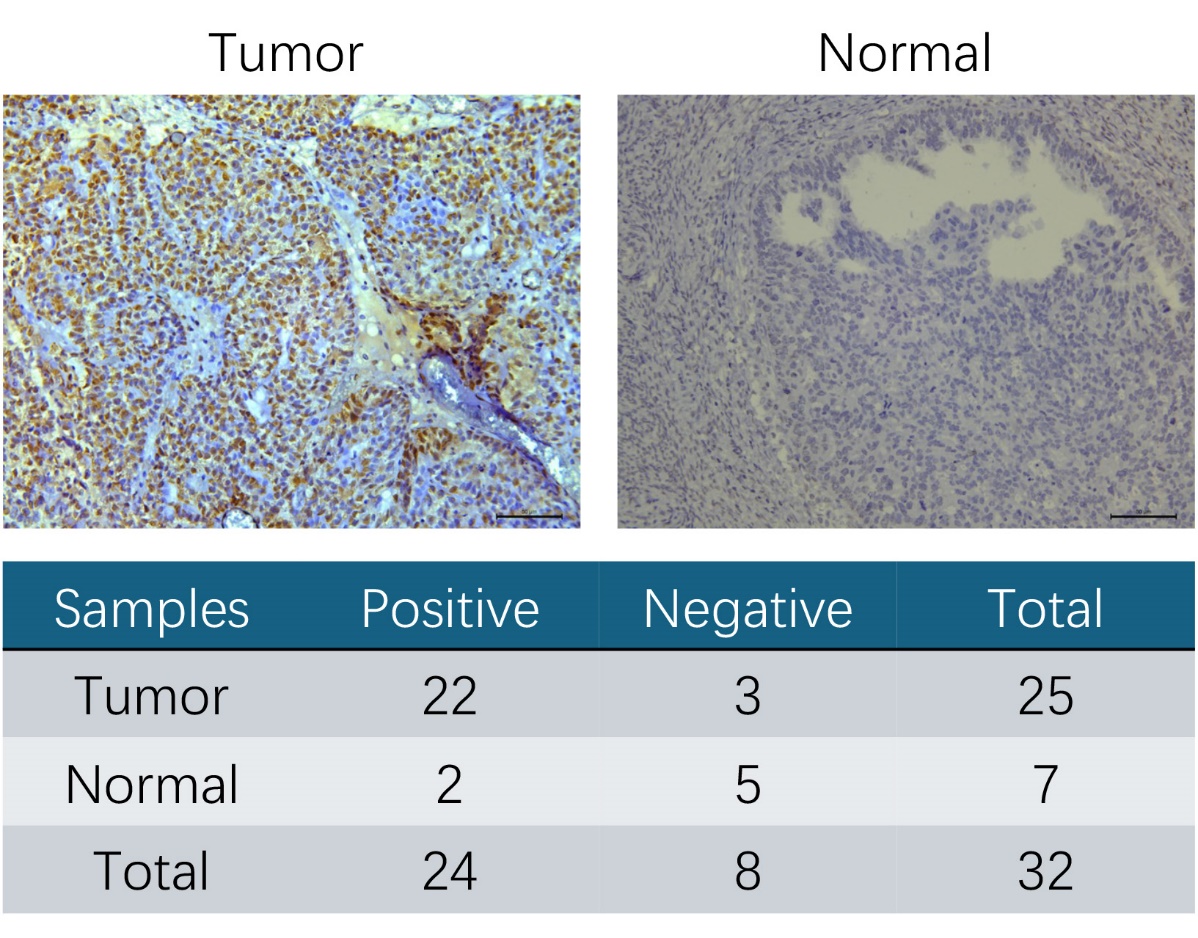


**Fig. S1. FZD5 expression in OC specimens.** FZD5 expression in 25 OC specimens and 7 normal ovarian tissues detected by immunohistochemistry; scale bar: 50 μm. *P* = 0.0048 by Fisher’s exact test.


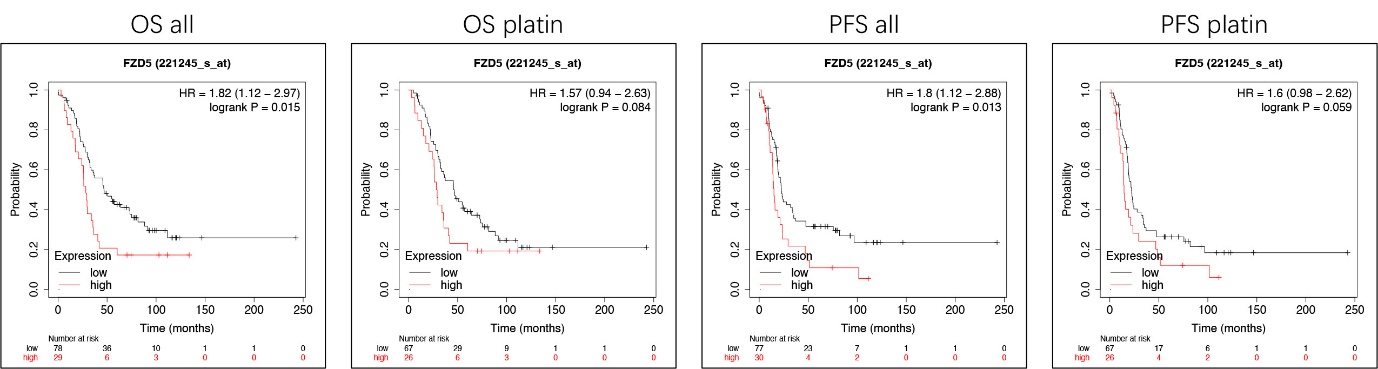


**Fig. S2. Survival analysis.** Correlation of *FZD5* with overall survival (OS) and progression-free survival (PFS) in OC analyzed using GSE26193 database at Kaplan-Meier Plotter website (https://kmplot.com/analysis/). “all”: all patients; “platin”: patients with platin treatment.

**
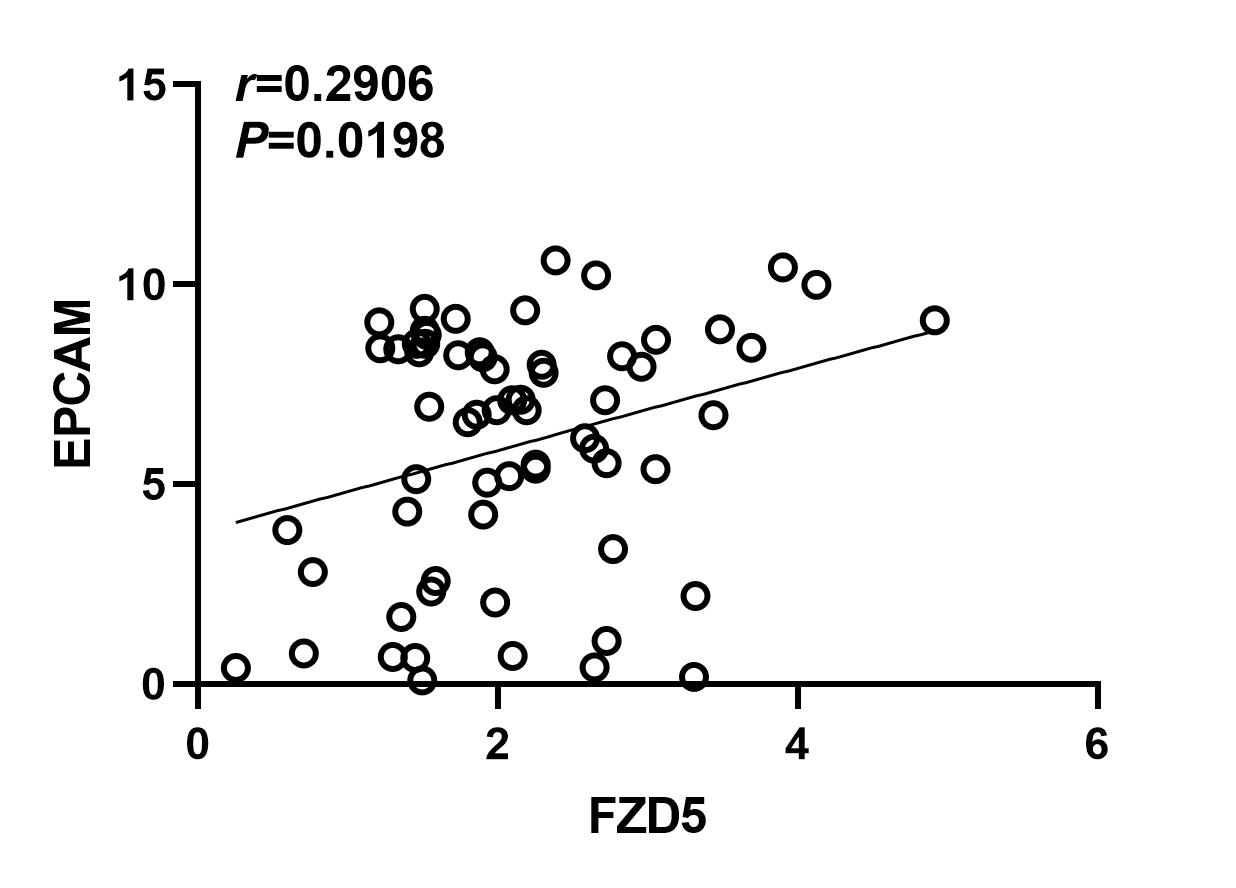
**

**Fig. S3. CCLE database analysis.** *FZD5* mRNA levels are positively correlated with those of *EPCAM* in OC.


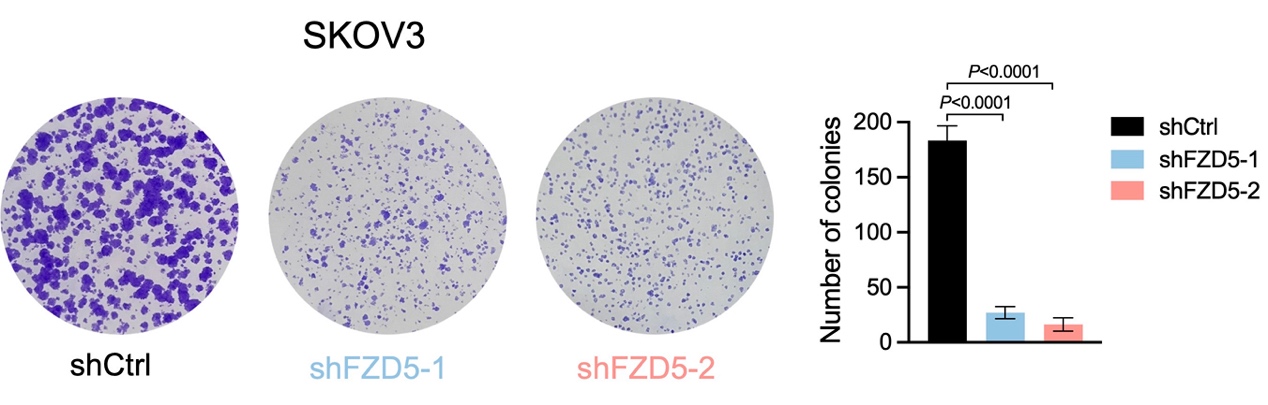


**Fig. S4. FZD5 knockdown inhibits SKOV3 cell growth.** Growth of SKOV3 cells determined by colony formation assay (mean ± SD, n = 3).


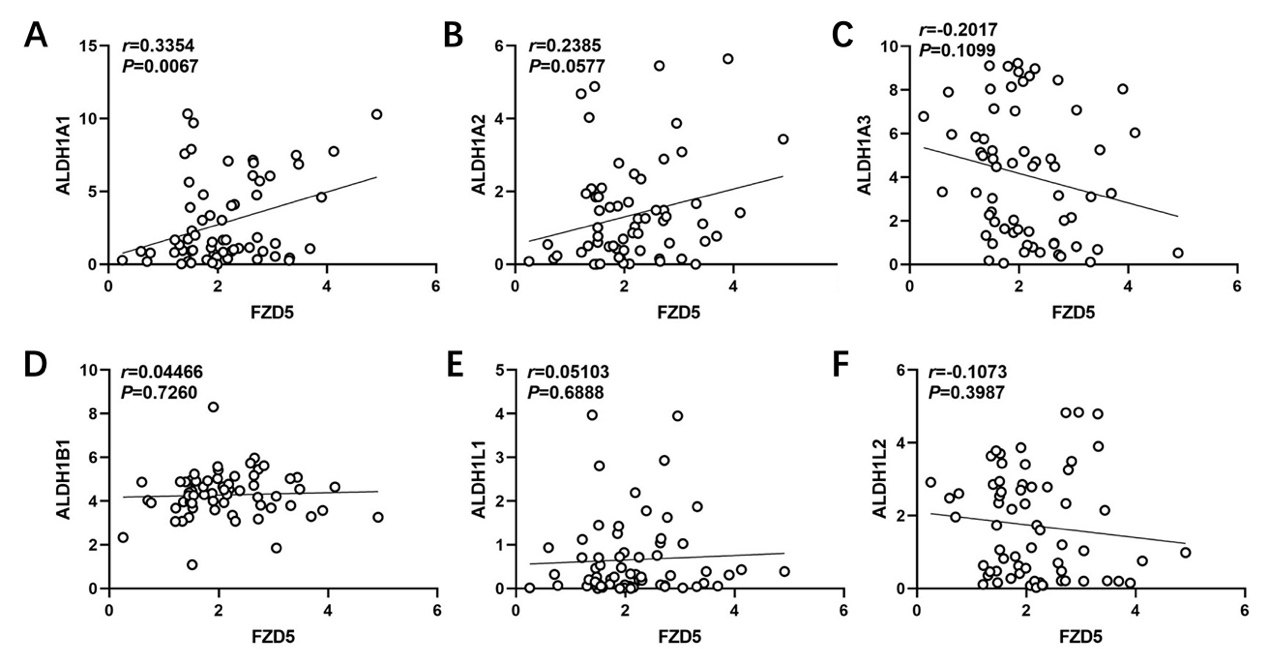


**Fig. S5. CCLE database analysis.** *FZD5* mRNA levels are positively correlated with those of *ALDH1A1*, but not with those of *ALDH1A2*, *ALDH1A3*, *ALDH1B1*, *ALDH1L1*, and *ALDH1L2* in OC.


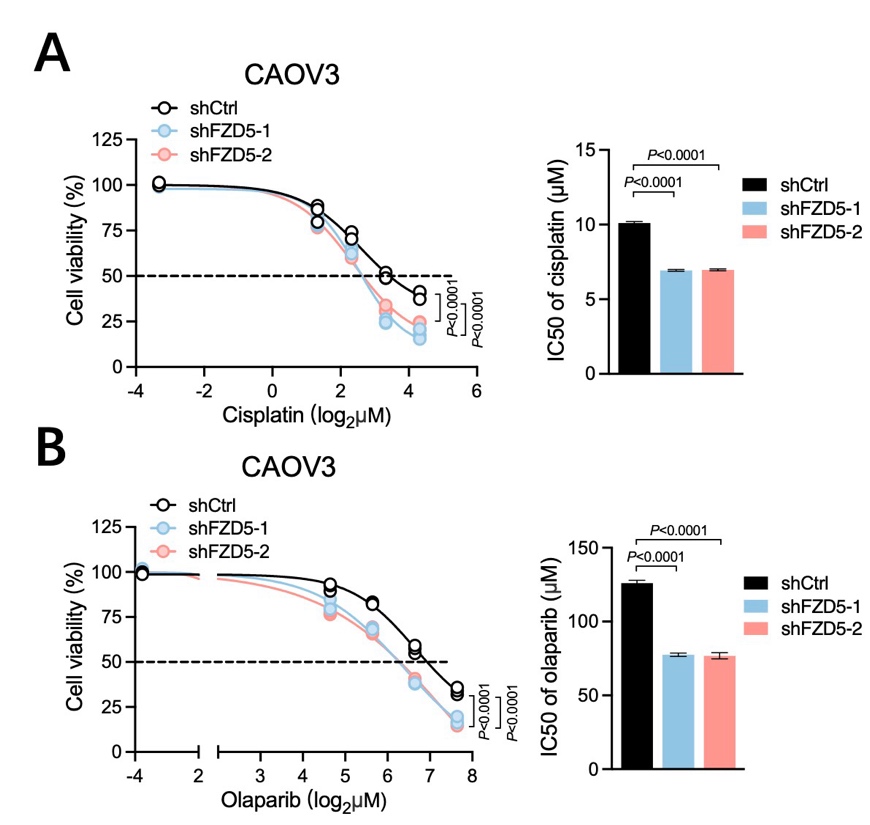


**Fig. S6. FZD5 knockdown sensitizes CAOV3 cells to chemotherapy A** Viability of CAOV3 cells after treatment with cisplatin analyzed by CCK8 assay (mean ± SD, n = 3). **B** Viability of CAOV3 cells after treatment with olaparib analyzed by CCK8 assay (mean ± SD, n = 3).


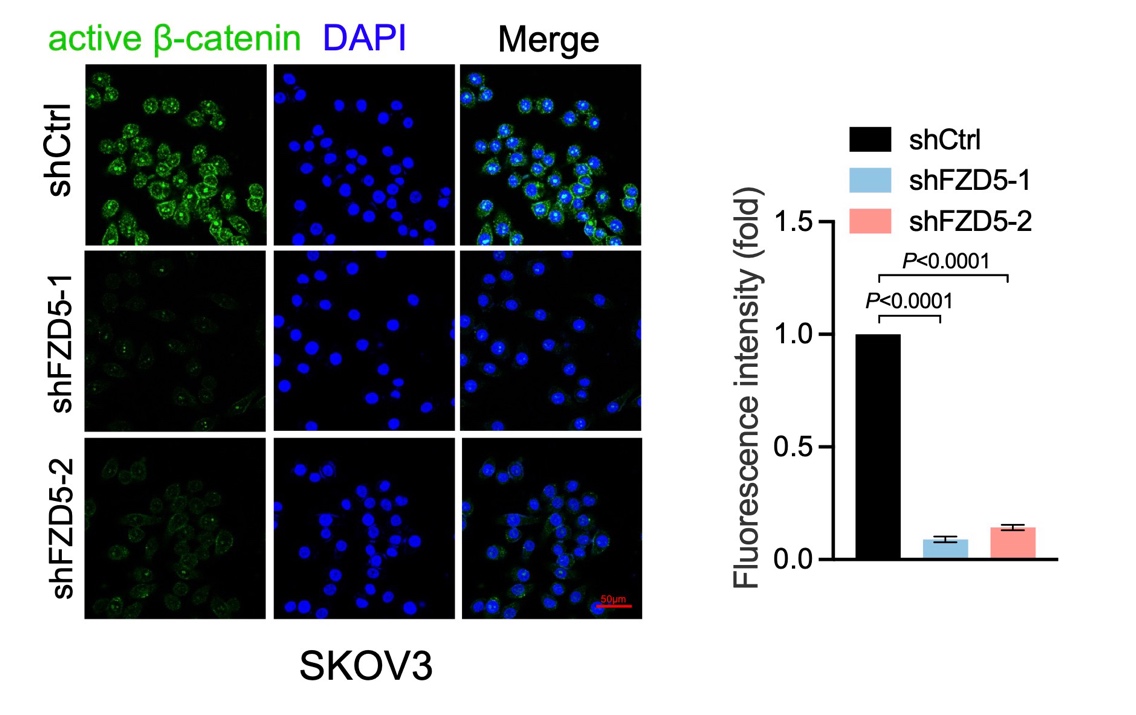


**Fig. S7. FZD5 knockdown reduces active β-catenin expression.** Active β-catenin expression in SKOV3 cells detected by immunofluorescence staining; scale bar: 50 μm.


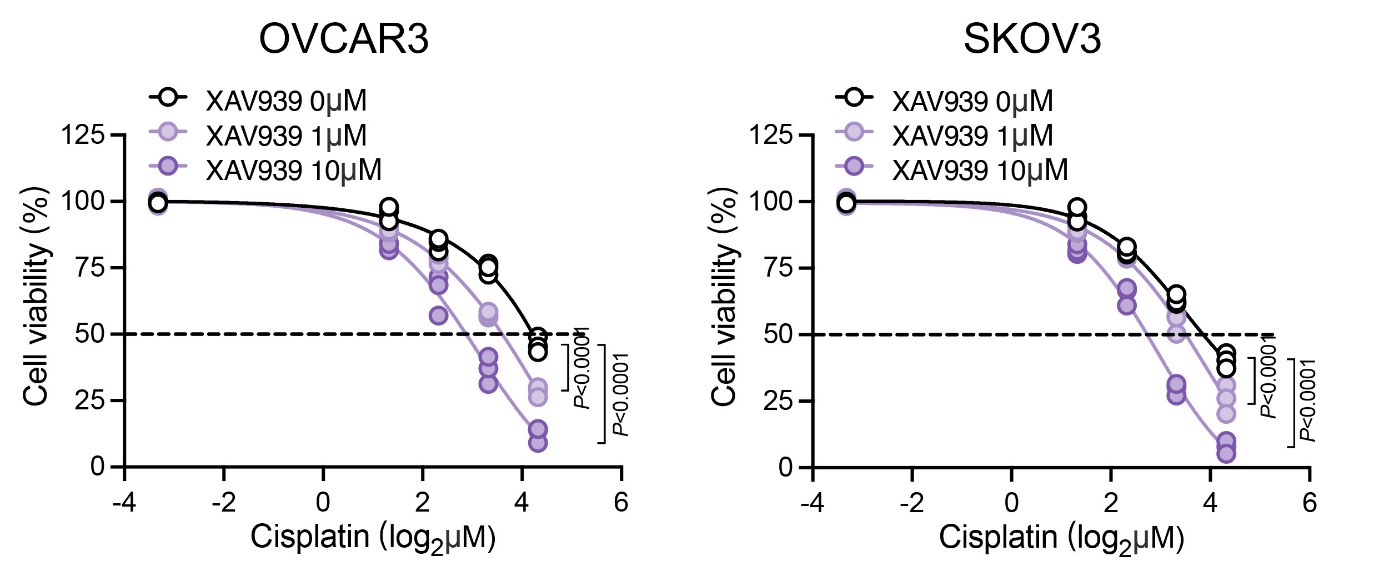


**Fig. S8.** **Treatment with XAV939 sensitizes OVCAR3 and SKOV3 cells to cisplatin.** Viability of OVCAR3 and SKOV3 cells after treatment with XAV939 and cisplatin analyzed by CCK8 assay (mean ± SD, n = 3).


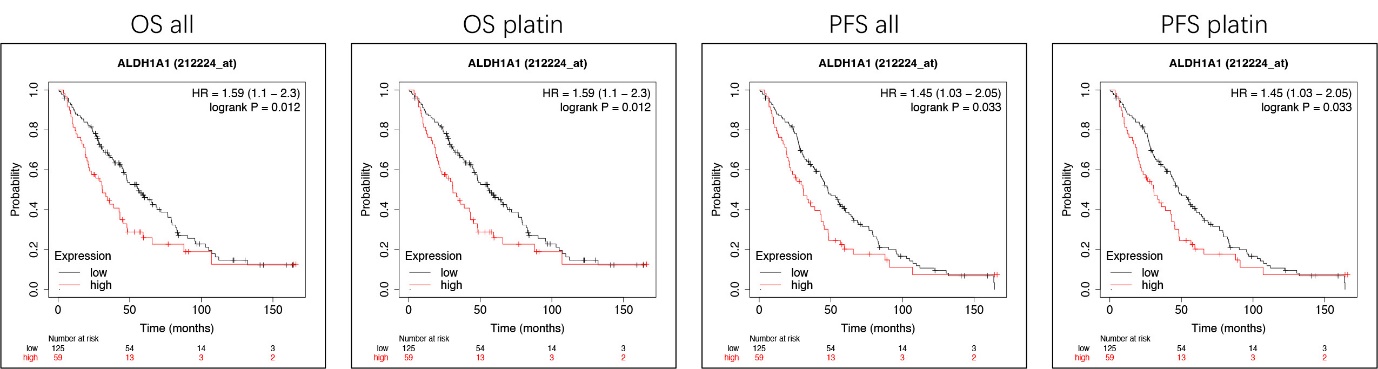


**Fig. S9. Survival analysis.** Correlation of *ALDH1A1* with OS and PFS in OC analyzed using GSE26712 database at Kaplan-Meier Plotter website. “all”: all patients; “platin”: patients with platin treatment.
